# Little Between-Region and Between-Country Variance When People Form Impressions of Others

**DOI:** 10.1177/09567976211019950

**Published:** 2021-11-02

**Authors:** Neil Hester, Sally Y. Xie, Eric Hehman

**Affiliations:** Department of Psychology, McGill University

**Keywords:** impression formation, person perception, cross-cultural psychology, face perception, geographical analysis, open data, preregistered

## Abstract

To what extent are perceivers’ first impressions of other individuals dictated by cultural background rather than personal idiosyncrasies? To address this question, we analyzed a globally diverse data set containing 11,481 adult participants’ ratings of 120 targets across 45 countries (2,597,624 total ratings). Across ratings of 13 traits, we found that perceivers’ idiosyncratic differences accounted for approximately 29% of variance and impressions on their own and approximately 16% in conjunction with target characteristics. However, country- and region-level differences, here a proxy for culture, accounted for 3.2% on average (i.e., both alone and in conjunction with target characteristics). We replicated this pattern of effects in a preregistered analysis on an entirely novel data set containing 7,007 participants’ ratings of 100 targets across 41 countries (24,886 total ratings). Together, these results suggest that perceivers’ impressions of other people are largely dictated by their individual characteristics and local environment rather than their cultural background.

To what extent are perceivers’ impressions of other individuals dictated by cultural background rather than personal idiosyncrasies? We used a first-of-its-kind globally diverse data set to examine to what extent higher-order culture—operationalized as region and country of residence—contributes to variation in first impressions of faces. We then conducted a preregistered replication of our findings using an independent data set.

## Sources of Variance in Impression Formation

Perceivers’ impressions are influenced by myriad factors: characteristics of the target, characteristics of the perceiver, and interactions between target and perceiver characteristics (referred to in this article as “perceiver-by-target interactions”; Freeman & Ambady, 2011; Hehman et al., 2017; Kenny & Albright, 1987; Kunda & Thagard, 1996; Todorov et al., 2015). How target characteristics such as facial features influence impressions is the best documented of these three sources (Hehman et al., 2019); hundreds of studies have investigated how specific facial features or other physical characteristics give rise to impressions of attractiveness, trustworthiness, dominance, and other traits (Hehman et al., 2014; Holzleitner et al., 2019; A. L. Jones & Jaeger, 2019; Oosterhof & Todorov, 2008; Todorov et al., 2015; Vernon et al., 2014). This body of work is framed by theories about why target appearance influences impressions, such as the *overgeneralization hypothesis* (Zebrowitz et al., 2003) and evolutionary theories of sexual selection (Thornhill & Gangestad, 1999).

Perceiver characteristics, though less understood, are also central to modern models of social cognition (Brewer & Kramer, 1985; Bruce & Young, 1986; Fiske & Neuberg, 1990; Freeman & Ambady, 2011; Kenny & Albright, 1987; Kunda & Thagard, 1996). Perceiver characteristics consist of any way in which one perceiver differs from another. Differences might be at the trait level, such as personality or concept knowledge, or at the state level, such as affective state or surrounding environment. Indeed, recent work has shown that people differ in their beliefs about trait cooccurrence (e.g., “How friendly is someone who is intelligent?”), which explains considerable perceiver-level variation in first impressions (Stolier et al., 2018, 2020). Perceivers forming impressions are not blank canvases onto which targets project impressions; instead, perceivers actively interpret their world through individual lenses.

Finally, perceiver-by-target interactions describe when impressions depend on features of both the perceiver and the target. This might include differences in trait ratings from stereotypes (e.g., beliefs that Black people are athletic) as well as from idiosyncratic links between features and traits (e.g., finding red hair attractive). These perceiver-by-target interactions are central to intergroup research, in which individuals in different groups differentially evaluate stimuli that vary by race, gender, or other identities. For example, perceivers higher in ambivalent sexism perceive men in egalitarian (rather than stereotypic) relationships as less warm and competent (McCarty & Kelly, 2015). Dynamic models of impression formation map out how target and perceiver characteristics continually interact across multiple levels of processing during impression formation (Freeman et al., 2020; Kunda & Thagard, 1996).

## Differences in the Importance of Variance Sources

Despite the historical focus on target-level variance, recent work has found that perceiver characteristics and perceiver-by-target interactions each play a larger role in overall first impressions (20–25% of the variance) than target characteristics do (10–15% of the variance; Hehman et al., 2017; Hönekopp, 2006; Xie et al., 2019). In this work, cross-classified multilevel models were used to decompose impressions into variance attributable to target characteristics, perceiver characteristics, and perceiver-by-target interactions (Kenny et al., 2006; Raudenbush & Bryk, 2002). Understanding the relative contribution of different sources of impression variance is critical to impression-formation theory. As a parallel, epidemiologists cannot effectively understand the dangers of a virus without knowing how much genetics and experience (i.e., nature vs. nurture), as well as their interaction, uniquely contribute to individual susceptibility. Similarly, to understand the extent to which perceiver- and target-level factors influence our impressions is to better understand the processes by which perceivers form impressions (see Hehman et al., 2017).

**Statement of Relevance**

For some time, researchers have investigated how people form first impressions of others. For example, researchers are interested in why people tend to perceive certain kinds of faces as more attractive or trustworthy than others (differences between targets), as well as why certain people tend to perceive faces as more or less attractive or trustworthy overall (differences between perceivers). One outstanding question about first impressions is how much first impressions vary across different cultures. In this study, we examined over 2.5 million face ratings across 57 different countries (which represent different cultural settings) to measure how much people’s first impressions vary across these different countries. Although many researchers have presumed that there is considerable variation in first impressions across cultures, we found that cultural setting explains much less variability in first impressions than do perceivers’ idiosyncrasies and targets’ characteristics.

Characteristics of the perceiver, both in the form of perceiver variance and perceiver-by-target interactions, thus account for considerable variance in any given impression. Previous research has quantified the extent of their contribution. However, it is unclear exactly which perceiver characteristics are important for guiding impression formation, because how one perceiver differs from another is so broad. Is the locus of this perceiver variability in the individual? Differences in how perceivers evaluate the same target may arise from idiosyncratic factors, such as personal interests, experiences, and beliefs. This is at least partly the case, as demonstrated by perceiver-level variability in ratings constrained to a single country (Hehman et al., 2017).

Alternatively, the locus could be the broader context in which individuals are embedded, such as cultural beliefs that cluster as a function of one’s country or global region. Because different perceivers are in different cultures when forming impressions, previous research would have identified effects of the broader environment as perceiver-level effects when in reality the true source of variance is not in the perceiver but instead in the broader culture and location. These higher-order clusters could potentially account for a large proportion of what has previously been identified as perceiver-level variance.

Cross-cultural research has shown important higher-order differences in first impressions (e.g., Birkás et al., 2014). Furthermore, work has shown that the factor structure of both trustworthiness and dominance in face perception (B. C. Jones et al., 2021) varies considerably as a function of culture, suggesting that region- and country-level cultural differences might play a large role in shaping impressions. However, other work comparing two specific cultures has found limited cultural variation in both the factor structure (Sutherland et al., 2018) and cultural variability (Zebrowitz et al., 2012) of first impressions. The present work contributes to the ongoing discussion regarding culture and first impressions by measuring cultural variability using a large and geographically diverse data set.

## The Present Research

Across a broad array of domains—social cognition, social perception, person perception, and others—researchers use trait impressions to understand how humans perceive others. Although it is important to disentangle idiosyncratic perceiver factors from systematic cultural factors in impression formation, doing so requires numerous perceivers within numerous cultures, which presents a challenge. We capitalized on a unique data set of 2,597,624 trait ratings from 45 countries (B. C. Jones et al., 2021) to compare the role of between-target, between-perceiver, and between-culture differences (operationalized as country and region) in first impressions. We solidified the value of our findings by conducting a preregistered replication of our study using a second data set provided by one of the reviewers of the manuscript (Zickfeld et al., 2021).

## Study 1

### Method

#### Participants

**Fig. 1.**
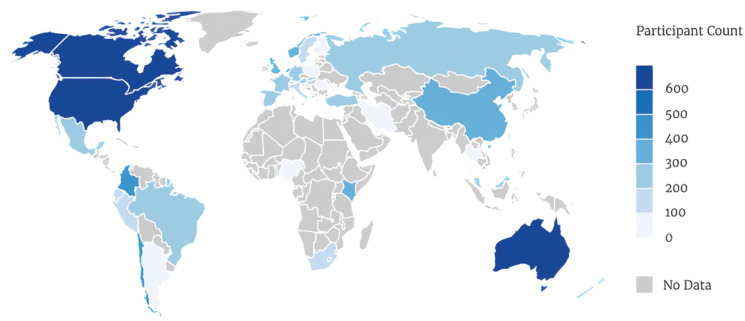
Distribution of participants across the 45 countries included in Study 1.

We analyzed the data set generated by the Psychological Science Accelerator 001 (PSA-001; B. C. Jones et al., 2021; Moshontz et al., 2018), a collaboration between 117 labs around the world to test the universality of the proposed basic dimensions of face perception (Oosterhof & Todorov, 2008). We followed their preregistered data-cleaning procedure, which retained 11,481 participants (69.6% women, 29.7% men, 0.7% other; mean age = 22.6 years), nested within 45 countries, nested within 11 regions (see Table 1 for a list of countries and regions as defined by PSA-001, and see Fig. 1 for a participant breakdown by country). Thus, we operationalized cultural variability as between-country and between-region variability. For more details on the selection of countries and the division into regions, see B. C. Jones et al. (2021).

**Table 1.** Countries by Region in Study 1, as Reported by Psychological Science Accelerator 001

World region	Countries within region
Africa (*n* = 520)	Kenya, Nigeria, South Africa
East and Southeast Asia (*n* = 780)	China, India, Malaysia, Taiwan, Thailand
Australia and New Zealand (*n* = 1,044)	Australia, New Zealand
Central America and Mexico (*n* = 338)	El Salvador, Mexico
Eastern Europe (*n* = 809)	Hungary, Lithuania, Poland, Russia, Serbia, Slovakia
Middle East (*n* = 503)	Iran, Israel, Turkey
United States and Canada (*n* = 3,312)	Canada, United States
Scandinavia (*n* = 653)	Denmark, Finland, Norway, Sweden
South America (*n* = 1,388)	Argentina, Brazil, Chile, Colombia, Ecuador
United Kingdom (*n* = 361)	England, Scotland, Wales
Western Europe (*n* = 1,862)	Austria, Belgium, France, Germany, Greece, Italy, The Netherlands, Portugal, Spain, Switzerland

One challenge of global data collection is accommodating various languages. Participants were given dictionary definitions for 12 of the 13 adjectives collected in order to mitigate the possibility that linguistic differences were responsible for variation (*dominant* was the exception, instead being defined as “strong, important”; B. C. Jones et al., 2021). To consider language as an alternate proxy for culture, we provide secondary analyses clustering by language rather than country and region.

#### Procedure

Each participant was randomly assigned to rate faces on one of 13 traits commonly used in person-perception research: *aggressive*, *attractive*, *caring*, *confident*, *dominant*, *emotionally stable*, *intelligent*, *mean*, *responsible*, *sociable*, *trustworthy*, *unhappy*, or *weird*. Participants completed 240 trials in which they rated neutrally posed faces on a 7-point scale (from *not at all* to *very*) for the assigned trait. The 240 trials were divided into two 120-trial blocks, and participants rated each face twice, enabling the partitioning of variance of the perceiver-by-target interactions from the residual (Hehman et al., 2017). The 120 faces were drawn from the Chicago Face Database (Ma et al., 2015) and evenly divided across ethnicity (Asian, Black, Latinx, White) and gender (female, male).

#### Analytic approach

We used multilevel models to calculate the amount of variance in trait ratings attributable to specific levels of clustering (e.g., perceiver, target, country, region). In these null or intercept-only models, participants’ ratings of stimuli on the dimension of interest (e.g., trustworthiness) served as the single dependent variable. The structure and size of the PSA-001 data allowed us to estimate four-level models for each trait: 2,597,624 trait ratings (Level 1) were cross-classified by 11,481 perceivers and 120 targets (Level 2). Perceivers were nested within 45 countries (Level 3) and countries within 11 regions (Level 4). Models were estimated using the R package *lme4* (Bates et al., 2015), and estimates from models that did not converge were confirmed in *brms* (Bürkner, 2017).

This model can be defined using four levels:

$$\mathrm{Level}\;1:Y_{i(jk)lm}=\pi_{0(jk)lm}+e_{i\left(jk\right)lm}$$

$$\begin{array}{l} \mathrm{Level}\;2:\pi_{0\left(jk\right)lm}=\beta_{000lm}+r_{0j000}+r_{00k00}+d_{0\left(jk\right)00}\\ \;\;\;\;\;\;+d_{00kl0}+d_{00k0m} \end{array}$$

$$\mathrm{Level}\;3:\beta_{000lm}=\gamma_{0000m}+r_{000l0}$$

$$\mathrm{Level}\;4:\gamma_{0000m}=\theta_{00000}+u_{0000m}$$

At Level 1, $Y_{i\left(jk\right)lm\;}$ is our dependent variable of interest: a rating on dimension *i* (e.g., trustworthiness) by perceiver *j* of target *k*, in which perceivers are nested within *l* countries within *m* regions. The intercept, $\pi_{0\left(jk\right)lm}$ , is the expected value of this rating, and the error term, $\;e_{ijk}$ , has its own associated variance, σ^2^. At Level 2 of the model, the intercept $\pi_{0\left(jk\right)lm}$ , is modeled as an outcome that varies across perceivers and targets, which allows the total variance of the model to be partitioned into that attributable to perceivers and targets. The group mean of perceiver ratings, $\beta_{000lm}$ , represents the expected value of the rating made by perceivers in country *l* (nested within region *m*) across all targets. The residual, $r_{0j000}$ , is the deviation of perceiver *j* from the mean score of their respective country (averaged across all targets), which has variance $\tau_{j00}$ . The other residual, $r_{00k00}$ , is the residual of target *k*, or the difference between the grand mean and the rating of target *k* averaged across all perceivers; these residuals have variance $\tau_{k00}$ . The random effect, $d_{0\left(jk\right)00}$ , represents the interaction between perceiver and target variance in the model and can be partitioned from error when a perceiver rates the same target at least twice (i.e., repeated measures within a perceiver and a target). The other two random effects are $d_{00kl0}$ , representing the interaction between target and country-level variance in ratings, and $d_{00k0m}$ , representing the interaction between target and region-level variance in ratings.

At Level 3 of the model, the expected value for the group mean, $\beta_{000lm}$ , is a function of the regional mean score, $\gamma_{0000m}$ (i.e., the average rating across countries within each region), plus each country’s residual from the mean rating of their region, $r_{000l0}$ , which has variance $\tau_{l00}$ . Finally, at Level 4, the expected value for the regional mean, $\gamma_{0000m}$ , is a function of the grand mean across all clusters (i.e., the average rating across all targets and perceivers across all countries and regions), plus each region’s residual from that grand mean, $u_{0000m}$ , with variance $\tau_{m00}$ .

Thus, we can estimate eight variance terms in the model: variance across perceivers, $\tau_{j00}$ ; variance across targets, $\tau_{k00}$ ; variance across countries, $\tau_{l00}$ ; variance across regions, $\tau_{m00}$ ; variance of the interaction between perceivers and targets, $\tau_{b00}$ ; variance of the interaction between targets and countries, $\tau_{c00}$ ; variance of the interaction between targets and regions, $\tau_{d00}$ ; and the Level 1 error term, σ^2^. Together, these terms comprise 100% of the variance in ratings on any dimension.

By looking at the size of each variance component relative to the total variance, we can calculate the proportions of variance that come from different elements of the model in an intraclass correlation coefficient (ICC; McGraw & Wong, 1996; Shrout & Fleiss, 1979). For example, target ICC is calculated as the proportion of variance attributable to perceiver characteristics:

$${\mathrm{ICC}}_{\mathrm{target}}=\frac{\tau_{k00}}{\tau_{k00}+\tau_{j00}+\tau_{l00}+\tau_{m00}+\tau_{b00}+\tau_{c00}+\tau_{d00}+\sigma^{2}}.$$

This approach descends from the social-relations model in dyadic impressions (Kenny et al., 2006). Using this approach, we can determine how much variance is attributable to individual factors (that do not correspond to location) as opposed to cultural factors (that do correspond to location).

Consider an example in which country ICC is .80. This result would indicate that 80% of the variance in a particular trait impression is due to between-country differences, suggesting that people in different countries were mostly drawing on shared cultural experiences when forming impressions. In contrast, if country ICCs are very low (e.g., .02), only 2% of the variance in trait impressions would be due to between-country differences, suggesting that other sources of variance were primarily driving the impressions. This latter situation highlights the importance of knowing the ICC. If an ICC is .02, no matter how many country-level variables are included in a model, they can together explain at most 2% of the variance in the trait impression. Such a situation would reveal that between-culture differences should perhaps not be a major focus of future research concerned with predicting and explaining people’s first impressions. Importantly, ICCs do not identify which variables are related to dependent variables but quantify only to what extent variance comes from different levels and, therefore, how to develop future theoretical models to best explain that variance.

Study 1 analyses were not preregistered. Instead, we tested an identical model across 13 different traits, providing multiple conceptual replications. We estimated ICCs for perceivers, targets, and perceiver-by-target combinations. Uniquely, we also estimated ICCs for countries, regions, country-by-target combinations, and region-by-target combinations. We used 95% confidence intervals (CIs) bootstrapped around each ICC (Xie et al., 2019). Together, these ICCs allowed us to quantify how much variance in trait ratings is attributable to between-country or between-region differences and to what extent a rating depended simultaneously on characteristics of the target and the country or region.

### Results

**Fig. 2.**
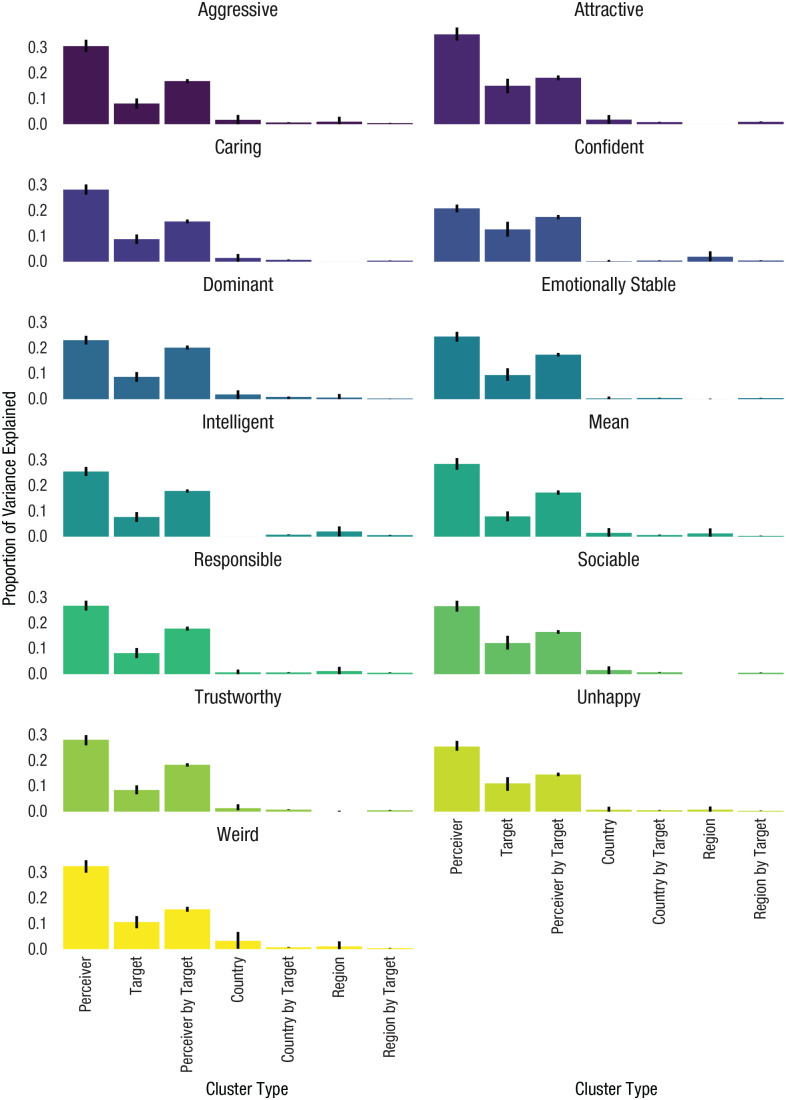
Intraclass correlation coefficients (ICCs) by cluster type in Study 1, separately for each of the 13 traits. Error bars represent 95% confidence intervals. Colors vary by trait and are provided for visual clarity.

Figure 2 displays the ICCs for all 13 traits. Although there was only minor variation in ICCs across traits (for full results, see https://osf.io/gry69/ under “Tables for All Models, Study 1”), they followed a consistent pattern. Across traits, perceiver differences accounted for the largest amount of variance (ICC: *M* = 29.1%; range = 21–35%), followed by perceiver-by-target differences (ICC: *M* = 15.6%; range = 15–20%) and target differences (ICC: *M* = 10.0%; range = 8–15%). Country and region differences, whether alone or interacting with the target, accounted for little variance in trait ratings (country ICC: *M* = 1.4%, range = 0–4%; region ICC: *M* = 0.7%, range = 0–2%; country-by-target ICC: *M* = 0.6%, range = 0–1%; region-by-target ICC: *M* = 0.5%, range = 0–1%).

The faces that participants had rated varied by ethnicity (i.e., Asian, Black, Latinx, White) and sex (i.e., female, male). One possibility was that while between-culture and between-region differences did not matter in the aggregate, perhaps their effect would be more substantial on specific ethnic and gender subgroups, given cultural variation in ethnic and gender stereotypes. To test this possibility, in supplementary analyses (see https://osf.io/gry69/), we fitted identical models for each subgroup (i.e., Asian female, Asian male, Black female, Black male, Latinx female, Latinx male, White female, White male). Yet between-culture and between-region differences were consistently not important for impressions of any subgroup. Results are available at https://osf.io/gry69/ under “Figures for Race-by-Gender Models.”

We additionally fitted models in which we replaced country (Level 3) and region (Level 4) with language (Level 3) to test whether language as a clustering variable yielded higher ICCs. It did not, yielding similarly low ICCs to country and region. These analyses are documented in the “Study 1 Syntax” provided at https://osf.io/gry69/.

Together, our results indicated that differences between country or region, which we used to operationalize high-level cultural differences, did not account for variance in trait ratings.

## Study 2

### Method

Study 2 was a preregistered conceptual replication of Study 1 using novel data from an investigation of the interpersonal effects of emotional crying across 41 countries (Zickfeld et al., 2021). The preregistration is available at https://osf.io/g59u6/. The key hypothesis in our preregistration was that region, region-by-target, country, and country-by-target clusters would account for no more than 8% of the total variance in ratings across all eight traits in the replication data set.

#### Participants

For our preregistered replication, we analyzed a data set from an investigation of the interpersonal effects of emotional crying across 41 countries (Zickfeld et al., 2021). We followed the authors’ preregistered data-cleaning procedure, which retained 24,886 trait ratings of 7,007 participants (68.9% women, 30.7% men, 0.7% other; mean age = 28.2 years), nested within 41 countries, nested within 11 regions (see Table 2 for a list of countries and regions). Traits were translated following the recommendations from PSA-001 (B. C. Jones et al., 2021).

**Table 2.** Countries by Region in Study 2, Categorized Using the Methodology of Psychological Science Accelerator 001

World region	Countries within region
Africa (*n* = 352)	Nigeria, South Africa
East and Southeast Asia (*n* = 1,192)	China, India, Japan, Malaysia, Philippines, Singapore, South Korea, Thailand
Australia and New Zealand (*n* = 156)	Australia, New Zealand
Central America and Mexico (*n* = 298)	Colombia, Mexico
Eastern Europe (*n* = 608)	Bosnia & Herzegovina, Croatia, Hungary, Poland, Serbia, Slovakia
Middle East (*n* = 1,141)	Israel, Pakistan, Turkey, United Arab Emirates
United States and Canada (*n* = 302)	Canada, United States
Scandinavia (*n* = 459)	Finland, Norway
South America (*n* = 488)	Argentina, Brazil, Chile, Peru
United Kingdom (*n* = 159)	Ireland, United Kingdom
Western Europe (*n* = 1,852)	Austria, France, Germany, Greece, The Netherlands, Portugal, Spain

#### Procedure

Participants completed four trials in which they rated neutrally posed faces from the Chicago Face Database (White, Black, Latinx, East Asian; Ma et al., 2015) and the Bogazici Face Database (Turkish; Saribay et al., 2018). Half of these faces were digitally edited to add tears (the facial expressions themselves were not edited) to address key hypotheses that motivated the original collection of the data. Furthermore, the study included manipulations of both situational valence and social context. For full details, refer to the article by Zickfeld and colleagues (2021). Although these manipulations might introduce additional variability on both the perceiver and target levels, they did not preclude estimations of variance at the country and region levels, which were the key estimates for our preregistered analyses.

For each face, participants provided ratings on a 7-point scale for the following adjectives: *attractive*, *capable*, *competent*, *dominant*, *friendly*, *honest*, *reliable*, and *warm*. Participants also provided other ratings unrelated to our hypotheses, which we have not included here.

#### Analytic strategy

We used the same analytic strategy as in Study 1, with the exclusion of the perceiver-by-target estimate of variance (two perceiver ratings of each target were necessary to dissociate this term from the error term).

### Results

**Fig. 3.**
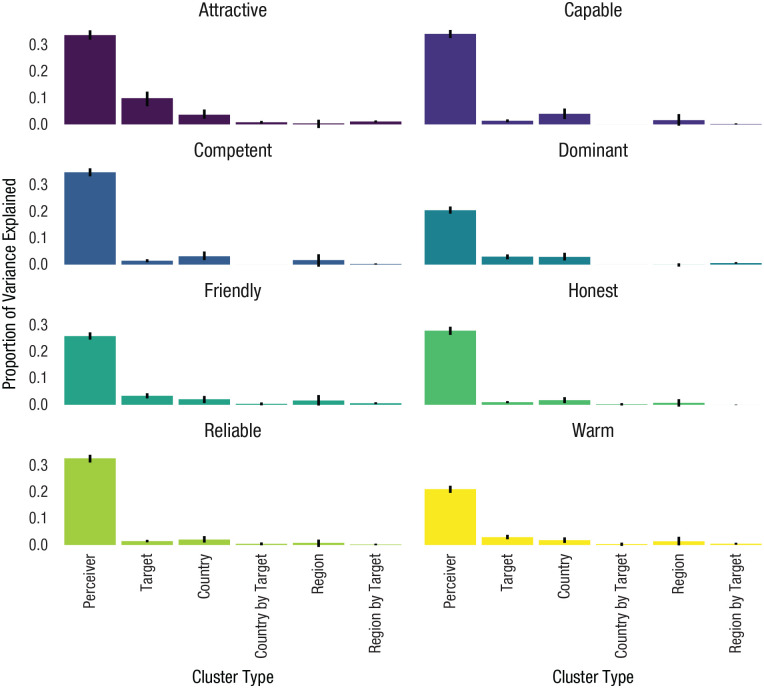
Intraclass correlation coefficients (ICCs) by cluster type in Study 2, separately for each of the eight traits. Error bars represent 95% confidence intervals. Colors vary by trait and are provided for visual clarity.

Figure 3 displays the ICCs for all eight traits. Although there was only minor variation in ICCs across traits (for full results, see https://osf.io/gry69/ under “Tables for All Models, Study 2”), they followed a consistent pattern. Across traits, perceiver differences accounted for the largest amount of variance (ICC: *M* = 28.9%, range = 21–35%). Target differences accounted for less variance than in the PSA-001 study (ICC: *M* = 3.1%, range = 1–9%); this difference might be due to the low number of target stimuli per participant. Country and region differences, whether alone or interacting with the target, accounted for little variance in trait ratings (country ICC: *M* = 2.7%, range = 2–4%; region ICC: *M* = 1.0%, range = 0–2%; country-by-target ICC: *M* = 0.3%, range = 0–1%; region-by-target ICC: *M* = 0.4%, range = 0–1%). We found evidence consistent with our preregistered hypothesis that country, region, country-by-target, and region-by-target clusters would account for less than 8% of the variance in face ratings (ICC: *M* = 4.4%, range = 3–6%). In general, results followed a pattern similar to that of Study 1.

## General Discussion

Analysis of over 2,500,000 trait ratings suggests that between-culture differences account for minimal variance in trait impressions inferred from faces. Consistent with previous work (Hehman et al., 2017; Hönekopp, 2006; Xie et al., 2019), our results showed that perceiver characteristics and perceiver-by-target interactions were larger sources of variance in impressions than target characteristics. Culture, operationalized as between-country and between-region variation, did not play a substantial role in the outcome of impression formation, accounting for at most 5% of the variance in any given trait in our sample (which, though the most diverse to date, still did not substantively sample from Africa, Asia, or from older-adult populations). Researchers wishing to examine between-culture variation in impressions might keep this upper threshold in mind.

The present results converge with recent research highlighting individual-centered variance in how impressions are formed. For example, research adopting a twin-study design partitioned the variability of personal environment and genetics in forming impressions of trustworthiness, attractiveness, and dominance. Results indicated that genetics explained little variability relative to one’s personal environment (Sutherland et al., 2020), which encompasses local factors related to one’s upbringing and one’s family and community environment, and are likely to drive the observed perceiver-level differences. Other work suggests that individuals’ conceptual trait spaces (i.e., the ways that different traits correlate with each other) are learned from actual personality structure in one’s environment, which may explain the similar structure observed in face, person-knowledge, and stereotype domains (Stolier et al., 2020). This work, together with the present results, supports the importance of individual variability in shaping the outcome of impression formation relative to genetic and cultural variability.

### Cultural heterogeneity in factor structure versus partitioned variance

The research generating this data found regional heterogeneity in the factor structure underlying impression formation (B. C. Jones et al., 2021). It is important to clarify that the present results are not at odds with this conclusion. Whereas we found that between-culture differences account minimally for variance in an impression of any single trait, work examining factor structure focuses on how different trait impressions covary. Identifying the source of variance in perceivers’ impressions is distinct from questions about structure. Although structure appears to vary regionally (B. C. Jones et al., 2021; Wang et al., 2019), variance in any individual’s trait ratings mostly arises from idiosyncratic perceiver and target differences.

This contrast implies that cross-cultural research—and any work that explores group differences—should treat questions about factor structure and questions about partitioned variance as theoretically distinct. One broad possibility is that the latent factor structure of impressions tends to vary by higher-order factors such as culture but that the variance in these impressions tends to vary by lower-order perceiver and target differences. In other words, it is possible that people’s concept knowledge of broad latent factors (i.e., what latent factors exist and what manifest variables reflect this latent factor) is more culturally determined, but the way that people infer a given trait from a stimulus is more individually determined—or, at least, is determined by a lower-order geography or culture (e.g., within-country regional units). Better understanding of this distinction is essential for forming domain-general theories of social perception that simultaneously discuss both factor structure and individual variance (Freeman et al., 2020).

### Limitations

Our conclusions rely on several assumptions. One is that participants in each country are representative of the way in which impressions are formed in that country. For example, the preponderance of undergraduate participants may make the data set nonrepresentative to such a degree that we failed to capture large amounts of cultural variation. It is likely that this feature of the data produces a conservative estimate of the effect of culture. However, it seems unlikely that young participants are unembedded in their countries’ or regions’ cultures to such a degree that they wholly obscure cultural effects. Further, at least in Western samples, variance estimates from undergraduates match those of the broader population (Hehman et al., 2017).

We also assumed that effects generalize to impression formation broadly, rather than only to this commonly used subset of 13 trait impressions (Oosterhof & Todorov, 2008). The stimuli also do not exhaustively represent the diverse populations by whom they were rated and demonstrate only neutral expressions. Future research might examine whether our results hold for different, more dynamic, and less controlled stimuli. Furthermore, we recognize that the samples in both studies drew from African and Asian countries in limited ways, as shown in Figure 1. The omission of these regions constrains our claims that these results generalize globally.

Finally, the present research operationalizes culture as between-country and between-region variation. Culture can vary dramatically by smaller intracountry units, and any intracountry cultural variation would be missed by the present models. Intracultural variation certainly exists for some individual differences. For example, U.S. states vary on Big Five personality traits (Rentfrow et al., 2008). Furthermore, because people travel and relocate, region and country as operationalizations of culture will include some measurement error that might lead to underestimation of country- and region-level effects. Finally, culture defined in other ways—such as rural–urban, liberal–conservative, or poor–rich—might still meaningfully impact impression formation. Future research could test whether culture defined in these ways reveals meaningful variation not captured here.

### Conclusion

For any one impression that a perceiver forms of a static face, higher-order cultural factors (i.e., those organized by country or region) play a relatively small role in what this impression will be, relative to personal idiosyncrasies or low-order cultural factors. The present results suggest that the most universal aspect of first impressions is their variability across individual perceivers and targets, regardless of location or culture.

## Transparency

*Action Editor:* Patricia J. Bauer

*Editor:* Patricia J. Bauer

*Author Contributions*

N. Hester and E. Hehman conceived the research and designed the methodology. N. Hester curated the data. N. Hester and S. Y. Xie analyzed the data. N. Hester wrote the original draft of the manuscript; all authors reviewed and edited the manuscript and approved the final version for submission.

**Open Practices:** All data used for Study 1 were obtained from Psychological Science Accelerator 001 and are available at https://osf.io/87rbg/. All data for Study 2 were obtained from the study by Zickfeld and colleagues (2021) and are available at https://osf.io/fj9bd/. The code and supplemental material for the studies reported in this article have been made publicly available via OSF and can be accessed at https://osf.io/gry69/. The design and analysis plan for Study 2 were preregistered at https://osf.io/g59u6/. This article has received the badges for Open Data and Preregistration. More information about the Open Practices badges can be found at http://www.psychologicalscience.org/publications/badges.
